# Lightweight and Energy-Aware Monocular Depth Estimation Models for IoT Embedded Devices: Challenges and Performances in Terrestrial and Underwater Scenarios †

**DOI:** 10.3390/s23042223

**Published:** 2023-02-16

**Authors:** Lorenzo Papa, Gabriele Proietti Mattia, Paolo Russo, Irene Amerini, Roberto Beraldi

**Affiliations:** Department of Computer, Control and Management Engineering, Sapienza University of Rome, Via Ariosto 25, 00185 Rome, Italy

**Keywords:** depth estimation, embedded devices, energy, real-time, underwater

## Abstract

The knowledge of environmental depth is essential in multiple robotics and computer vision tasks for both terrestrial and underwater scenarios. Moreover, the hardware on which this technology runs, generally IoT and embedded devices, are limited in terms of power consumption, and therefore, models with a low-energy footprint are required to be designed. Recent works aim at enabling depth perception using single RGB images on deep architectures, such as convolutional neural networks and vision transformers, which are generally unsuitable for real-time inferences on low-power embedded hardware. Moreover, such architectures are trained to estimate depth maps mainly on terrestrial scenarios due to the scarcity of underwater depth data. Purposely, we present two lightweight architectures based on optimized MobileNetV3 encoders and a specifically designed decoder to achieve fast inferences and accurate estimations over embedded devices, a feasibility study to predict depth maps over underwater scenarios, and an energy assessment to understand which is the effective energy consumption during the inference. Precisely, we propose the MobileNetV3${}_{S75}$ configuration to infer on the 32-bit ARM CPU and the MobileNetV3${}_{LMin}$ for the 8-bit Edge TPU hardware. In underwater settings, the proposed design achieves comparable estimations with fast inference performances compared to state-of-the-art methods. Moreover, we statistically proved that the architecture of the models has an impact on the energy footprint in terms of Watts required by the device during the inference. Then, the proposed architectures would be considered to be a promising approach for real-time monocular depth estimation by offering the best trade-off between inference performances, estimation error and energy consumption, with the aim of improving the environment perception for underwater drones, lightweight robots and Internet of things.

## 1. Introduction

The perception of the surrounding environment and the overall energy consumption are two crucial characteristics for the design of IoT-enabled robotic systems that have to interact and move in both terrestrial and underwater scenarios [1]. Indeed, these devices are generally powered with batteries, and therefore, their lifespan can be increased if the software is designed to be energetically efficient. In our specific scenario, this comprehends the designing of deep models which require the least possible amount of energy during the inference phase. Environment perception is commonly performed through passive systems such as LiDAR and sonar devices that can detect and measure the presence and position of objects or obstacles in the surrounding environment through the emission of a light or sound signals. However, such devices are often bulky and expensive, making them unsuitable for many possible application scenarios, such as mounted on small robots, underwater drones, or used in impervious locations. Moreover, those systems, due to their light design, are usually characterized by limited computational memory and energy availability. Under these constraints, camera-based measurements and deep learning (DL) algorithms might be viable solutions.

The monocular depth estimation (MDE) is a DL task where the depth related to the scene is estimated through a single RGB image. In recent computer vision and deep learning trends, researchers focus their attention on achieving the highest estimation accuracy without taking into account the computational effort and the energy consumption required to run developed models in real-world vision applications such as small robots, aerial and underwater drones. In the MDE task, this tendency can be noticed in recent proposed works such as [2,3,4,5]. Those algorithms typically infer in the cloud or on dedicated servers without considering possible low-resource hardware constraints. On the other side, some recent DL studies such as [6,7] are going against this paradigm, analyzing the Edge TPU’s capabilities in deep learning tasks. The embedded hardware is characterized by low-power consumption and limited memory capacity, acting as a performance bottleneck for DL-based techniques. Regarding monocular depth estimation, only a few works propose a solution for porting such complex tasks on low-resource platforms. There are two main approaches: [8,9,10] that focus on MDE on microcontroller and ARM-powered devices without taking into account the inference frequency and [11,12,13], which analyze the inference performances of MDE on low-power embedded GPUs. Furthermore, MDE methods are usually trained in supervised learning strategies on indoor and outdoor terrestrial datasets such as [14,15]. Although estimating depth and the distances to objects could be a critical feature for autonomous underwater systems, due to the lack of labeled data, only a few works such as [16,17,18,19] propose qualitative underwater depth measurements by relying on color restoration and the correspondence between depth and visual-style levels.

This work investigates the previously mentioned issues by choosing benchmark hardware components, an ARM CPU and an Edge TPU, which are widely employed in lightweight robots and AI-accelerator platforms (e.g., Coral Dev Board). Due to the constraints introduced by the selected embedded hardware, we propose two models for two different precision data types, i.e., 32-bit floating points to infer on the ARM CPU and 8-bit integers for the Edge TPU.

The contributions of this paper can be summarized as follows:

- We propose two lightweight MDE deep learning models that are able to achieve accurate estimations and fast inference frequencies on two embedded IoT devices;
- We conduct a feasibility study of such architectures in underwater scenes;
- We conduct a series of energy measurements during the inference of the proposed models to statistically prove their effective difference in the mean average consumption.

The paper is organized as follows: Section 2 reviews some previous works related to lightweight MDE methods in terrestrial and underwater scenarios and to energy-oriented deep learning. Section 3 describes the proposed architectures, while in Section 4 are presented their implementation details and the hyperparameters settings. Section 5 shows the obtained results in the two analyzed scenarios. Section 6 presents a statistically validated energy assessment of all the considered models. Finally, the last Section 7 concludes our study with some considerations on the underwater setting, energy consumption impacts in deep learning, and possible future works.

## 2. Related Works

In this section, we report state-of-the-art-related works for terrestrial and underwater depth estimation, respectively, in Section 2.1 and Section 2.2, as well as previous studies on energy-oriented deep learning models in Section 2.3.

### 2.1. Lightweight Terrestrial Monocular Depth Estimation

Monocular depth estimation methods are usually trained on terrestrial images using large-scale datasets such as the NYU Depth V2 [14] and the KITTI [15] datasets. Under resource-constrained scenarios, few works have been proposed so far to port such complex tasks on low-resource platforms. Previous research trends have mostly focused on two approaches based on these restricted assumptions. The first one directs its attention on microcontroller and ARM-powered devices without taking into account fast inference frequency performances. Poggi et al. [9] propose PyD-Net, a pyramidal feature extraction architecture to infer on low-energy devices. The model is aimed to overcome cutting-edge design difficulties, which are often deep and complicated, requiring dedicated hardware for their execution such as high-end and power-hungry GPUs. Peluso et al., on their side, propose [8,10]. The first work presents a framework for optimizing inference performance in order to produce a low-latency/high-throughput code. The system has been evaluated on the PyD-Net architecture, demonstrating good accuracy estimation with a number of resources compliant with ordinary CPUs. Furthermore, the second work builds on the previous one by presenting μPyD-Net, a model designed to tackle the MDE task under microcontroller computational power constraint and random access memory limitations. The authors also investigate the performance degradation caused by the quantization procedure, i.e., the model conversion from 32-bit to 8-bit precision data type.

The second trend of research investigates MDE inference performances on low-power GPUs. Spek et al. [13] propose CReaM, a model developed through a knowledge transfer strategy between a large supervisor model and the constrained one, to achieve accurate and real-time performances on the reference NVIDIA-TX2 GPU. Wofk et al. [12] present FastDepth, an architecture based on the MobileNet V2 encoder and a specifically designed decoder that achieves near real-time frequency performances on the NVIDIA-TX2 CPU and high frame rates on the considered GPU. Papa et al. [11] propose SPEED, a separable pyramidal pooling architecture designed to achieve real-time frequency performances on both cloud CPU, TPU, and edge devices, e.g., NVIDIA-TX1 GPU. The model makes extensive use of depthwise separable convolutions to achieve fast and accurate depth estimations.

Differently from these approaches, we propose to develop architectures that consider inference performances together with different precision data types, i.e., designing models capable of achieving real-time inferences on both 32-bit ARM CPUs and 8-bit Edge TPUs.

### 2.2. Underwater Depth Estimation

Compared with terrestrial monocular depth estimation, very few works focus on the estimation of depth in underwater environments due to the lack of training data. Moreover, due to the limited visibility and distortions introduced in underwater vision, researchers mainly focus on color restoration processes. Peng et al. [18] propose to estimate underwater depth distances using as features the image blurriness, where objects that are farther away appear more blurry than closer ones. Then, the estimated depth map is used to enhance respective RGB images using the image formation model. Drews et al. [19] present Underwater DCP, an improved Dark Channel Prior image restoration process for underwater scenarios. The approach computes colorized relative depth maps in order to extract geometrical information about the objects and, as a result, improves the visual quality of underwater images. Gupta et al. [17] propose a method composed of two connected dense-block-based auto-encoders to transform images from a hazy underwater domain to a hazy terrestrial (indoor) RGBD domain and vice versa. Ye et al. [16] propose a framework trained in an adversarial learning manner composed of two networks. The first network exploits a style adaptation model to learn style-level transformations to adapt terrestrial images to the style of the underwater domain, with the objective of rendering synthetic underwater images starting from terrestrial RGBD data. Then, the second network is used to jointly estimate the scene depth and correct the color from a single underwater image.

However, such methods are focused on closing the gap between underwater and terrestrial images, mainly proposing qualitative underwater depth estimations which rely on color restoration and on the correspondence between depth and visual-style levels. In contrast, we propose to shift the knowledge of the model from a terrestrial to an underwater scenario, exploiting color restoration processes to achieve effective and quantitative depth measurements.

### 2.3. Energy-Oriented Models

The energy footprint of the inference performed with deep learning models is not widely addressed in the literature. However, it becomes essential when models have to be installed in energy-constrained devices, which can be the typical usage scenario of lightweight models. Indeed, the preference for an energy-saver model is decisive in this case. Daghero et al., in [20], illustrate the most relevant techniques used for optimizing neural network models to support inference at the Edge. The techniques are divided into two main categories: the ones which are static and performed at the design time and the ones which are defined as dynamic since they depend on the input. However, the energy aspect, from a quantitative point of view and in terms of current or power, is not taken into consideration. Wang et al. in [21] propose a GPU implementation of RNN, which is based on a memristor technique. However, we assume the hardware architecture to be constrained, and we focus on the energy consumption of the specific models in the same hardware architecture. Lee et al. in [22] propose the logarithmic encoding of the weights in order to reduce the computational footprint of the model and, as a consequence, the energy efficiency, but the authors did not take into account the specific gain in terms of power consumption. Instead, in [23], the authors propose an energy-efficient toolkit for performing inference in Edge Computing. The toolkit is based on the “Fast Artificial Neural Network” (FANN) library to run inference in microcontrollers based on ARM CPUs; the authors also present power and energy studies of the proposed toolkit. However, in this paper, we focus on more powerful devices such as the Coral Dev Board, and we provide an energy assessment for models based on running with the TensorFlow Lite library. In [24], the authors propose a methodology for improving the energy efficiency of neural network models based on the possibility of re-using the computation, however, the energy assessment is simulated as a run in a microcontroller. Finally, Tasoulas et al., in [25], propose neural networks’ approximated multipliers with multiple accuracy levels at runtime. The approach has the objective of reducing the computational complexity of the Multiply-and-Accumulate (MAC) operation, which is at the core of neural networks’ inference process. However, the gain in terms of actual energy within a sample hardware is not taken into consideration, which is a part of the purpose of this work.

## 3. Proposed Method

**Figure 1:**
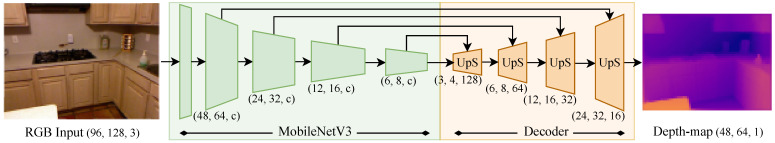
Graphical overview with respective input shapes of the proposed encoder–decoder structure. The number of channels (*c*) for MobileNetV3 and MobileNetV3${}_{LMin}$ are, respectively, [16,16,72,96]${}_{S75}$ and [16,64,72,240].

This section describes the MDE architectures developed to achieve the best trade-off between real-time frequency performance and estimation accuracy across multiple scenarios. We designed those models in compliance with two essential concepts: the *speed* to maximize the inference frequency on embedded devices, and the *robustness* to maximize the estimation accuracy across two datasets [14,26]. Our solution exploits an encoder–decoder model, similar to previous related works, such as [11,12]. In more detail, we perform an in-depth study on lightweight encoders pre-trained on ImageNet [27] to improve their generalization capabilities. A graphical overview of the proposed architectures is reported in Figure 1.

We chose MobileNetV3 [28] as the baseline encoder for further refinement. This fully convolutional network is built on a sequence of downsampling *inverted residuals blocks*, each one characterized by a Squeeze-and-Excite [29] block followed by a pointwise and a depthwise convolution. This procedure keeps a limited number of multiply and accumulate (MAC) operations, thus leading to significant speed improvements without introducing any substantial estimation error compared to other lightweight networks [30,31,32,33].

To tackle the MDE task under low resource constraints, we propose two configurations of the chosen baseline encoder, respectively, named MobileNetV3${}_{S75}$ and MobileNetV3${}_{LMin}$.

The MobileNetV3${}_{S75}$ model is the *Small (S)* configuration of the MobileNetV3 [28] with a reduction of 25% of the original convolutional filters while the MobileNetV3${}_{LMin}$ is based on the deeper *Large (L)* but *Minimalistic (Min)* configuration of the MobileNetV3. It is a variant of the original *Large* architecture where the Squeeze-and-Excite blocks are removed, Hard-Swish activations are replaced with ReLUs, and 5×5 convolutions are replaced with 3×3 ones. The MobileNetV3${}_{S75}$ model with its shallower design is designed to run on the 32-bit ARM CPU, while the deeper MobileNetV3${}_{LMin}$ architecture can be exploited on 8-bit Edge TPU devices. As will be shown in Section 5, the two encoders, based on different configurations of the same baseline architecture, are both able to achieve almost real-time performances and a notable estimation accuracy in their respective embedded hardware when compared with other lightweight backbones [28,30,32,33].

Furthermore, to maximize the estimation accuracy and reconstruction capabilities of the network, we designed from scratch a fully convolutional decoder that generalizes over the developed encoders. It is composed of a sequence of four cascaded up-sampling blocks (UpS) that increase the spatial resolution while merging the encoded features through a skip-connection to reconstruct the output depth map (see Figure 1). Each UpS, named TDSConv2D, is composed of two 3×3 convolutional operations, a transposed and a depthwise separable one interleaved by the skip-connection used to retrieve the image details from the encoder feature maps. This design allows for us to limit the number of computed operations, restricting the computational complexity while improving the overall inference frequency, as will be shown in Section 5.2.

## 4. Implementation Details

We trained the reported architectures with the same input–output resolution (respectively, 96×128 and 48×64) on the NYU Depth V2 [14] dataset with the official train-test split. We evaluated the generalization capabilites in underwater scenarios with respect to comparable state-of-the-art related models over the 57 stereo samples of the underwater SQUID [26] dataset. This study has been performed using TensorFlow 2 (https://www.tensorflow.org/, accessed on 12 February 2023) deep learning high-level API. Each compared model was initialized on ImageNet [27] pre-trained weights. ADAM [34] optimizer was used in all the experiments with the following setup: learning rate 0.0001, $\beta_{1}=0.9$, and $\beta_{2}=0.999$. We had set a batch size of 32 and trained the models for a total of 30 epochs on the chosen dataset. Once the training phase was completed, each model was converted and evaluated in TensorFlow Lite (https://www.tensorflow.org/lite, accessed on 12 February 2023), an open-source framework specifically designed to infer on embedded devices. Finally, as introduced in [35], to evaluate and compare the performance of proposed models, we considered the most commonly used metrics in MDE: the root-mean-square error (RMSE), the relative error (REL) and the accuracy value $\delta_{1}$. Their formal definitions are reported in Equations (1)–(3), where $p_{i}$ and $g_{i}$ are, respectively, the predicted depth map and its ground truth, while *P* denotes the total number of the evaluated pixels.

(1) $$RMSE=\sqrt{\frac{1}{\left|P\right|}\sum_{i\in P}\left|\right|p_{i}-g_{i}{\left|\right|}^{2}}$$

(2) $$REL=\frac{1}{\left|P\right|}\sum_{i\in P}\frac{|p_{i}-g_{i}|}{g_{i}}$$

(3) $$\delta_{1}=\frac{1}{\left|P\right|}\sum_{i\in P}\left(\gamma (p_{i},g_{i})\right)\;\;\mathrm{where}\;\;\gamma \left(p_{i},g_{i}\right)=\left\{\begin{array}{cc} 1 & \;\mathrm{if}\max\left(\frac{p_{i}}{g_{i}},\frac{g_{i}}{p_{i}}\right)<thr\\ 0 & \;\mathrm{otherwise} \end{array}\right.$$

In the last equation, thr is a threshold commonly set to 1.25.

Moreover, we compare the inference performances measured in frame-per-second (fps) using an Edge processing unit, the 4GB Google Coral Dev Board (https://coral.ai/products/dev-board/, accessed on 12 February 2023), a cheap and low-power embedded device equipped with a 32-bit ARM Cortex CPU and an 8-bit Edge TPU.

## 5. Results

This section compares the proposed architectures and the assessed encoders and decoders over different precision data types. The main results are obtained on the terrestrial dataset, while in the feasibility study, we analyzed the models’ estimation capabilites in underwater settings.

We report in Section 5.1 and Section 5.2 the individual contributions of the proposed encoders and the decoder, described in Section 3, while in Section 5.3 we analyze the accuracy and inference performances changing the input–output image resolution; in Section 5.4, we conduct the feasibility study to estimate depth maps over the underwater scenario.

### 5.1. Encoders

The first performed analysis is focused on the comparison of several lightweight pre-trained architectures such as [28,30,31,32,33] in order to choose the best encoder for our task. The estimation capabilites of those encoders with the proposed decoder structure are reported in Table 1; as can be noticed, the *Small* variant of the MobileNetV3 (Mob.NetV3${}_{S}$) obtains the highest frequency performance (close to 30 fps) on the CPU.

Based on the reported results, with the goal of achieving real-time inference performances, we chose as baseline encoders the *Small (S)* and *Large (L)* configurations of the MobileNetV3 (the last two rows of Table 1). Compared with the more accurate MobileNetV1, those models show a boost of 153% and 300% on the inference frequency at the expense of a slight increase in the RMSE equal to 12% and 17%, respectively.

**Figure 2:**
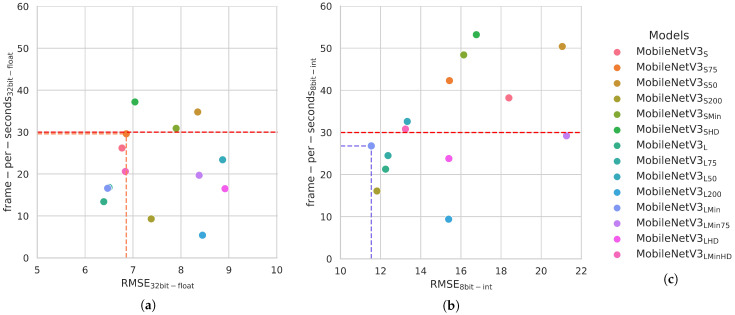
Graphical comparison between different MobileNetV3 configurations tested over the NYU Depth v2 dataset. The RMSE is reported in each graph in different ranges due to the respective error distribution and frame-per-seconds (fps). The red-dotted line is used to represent the real-time frame rate i.e., 30 fps, while the colored dotted segments in orange and light blue, respectively, are used to identify the best models. (**a**) The evaluated models for ARM CPU, 32-bit floating point precision. In orange, the best configuration, named MobileNetV3${}_{S75}$ (orange dot); (**b**) The evaluated models for Edge TPU, 8-bit integer precision. In light blue, the best configuration, named MobileNetV3${}_{LMin}$ (light-blue dot); (**c**) The list of the compared models.

As a second step, we focused on the optimization of the two baseline encoders adopting different configurations. A graphical comparison between the frame rates (fps) and the estimation error (RMSE) of the different configurations is reported in Figure 2; we used 30 fps as a reference value to identify the target (real-time) inference frequency, as seen in previous works [12,13].

Precisely, we compared multiple setups of the shallower MobileNetV3${}_{S}$ and deeper MobileNetV3${}_{L}$ configurations with the following modifications: reducing the number of convolutional filters by a specific percentage value, i.e., 50%, 75%, 200% on the encoder and 50% on the decoder, while fixing their size to 3×3 in the *Min* variant, instead of 5×5 used in the original model. We compared all the considered architectures in Figure 2. The names used for the different models are composed as follows: the name of the baseline backbone (Mob.NetV3) followed by the subscript *S* or *L* and the type of optimization applied. For example,  MobileNetV3${}_{SHD}$ is the MobileNetV3 in the *S* configuration with a reduction of 50% in the original convolution filters, whereas the MobileNetV3${}_{LMin75}$ is the *L* and minimalistic (Min) MobileNetV3 configuration with a reduction of 75% of the convolution filters. From this analysis, we identified the two best models named Mob.NetV3${}_{S75}$ (orange dot in Figure 2a) and Mob.NetV3${}_{LMin}$ (light-blue dot in Figure 2b), respectively, for the ARM CPU and Edge TPU.

The selected networks are able to achieve near real-time performances on both hardware, i.e., 29.6 fps on the CPU and 26.8 fps on the TPU, and an RMSE of 6.86 and 11.54 decimeters, respectively. Moreover, the increased depth estimation error obtained for the MobileNetV3${}_{LMin}$ on the Edge TPU is justified by the quantization operation, which compresses the model from 32-bit floating points to 8-bit integers. Finally, a complete overview of the results achieved by the two proposed models is reported in Table 2 while using the TDSConv2D decoder.

### 5.2. Decoder

Once the encoder configuration has been determined, we compared five different UpS blocks. The up-sampling operation has the fundamental role of progressively increasing the features resolution learned by the encoder while reconstructing the image details to obtain the final output depth map.

The detailed characteristics of each UpS are below reported:

- UpConv2D: a 2×2 up-sampling layer followed by a 3×3 convolution.
- UpDSConv2D: a 2×2 up-sampling layer followed by a 3×3 depthwise separable convolution.
- NNConv5 [12]: a 5×5 convolution followed by a 2×2 up-sampling with the nearest-neighbor interpolation.
- TConv2D: a 3×3 transposed convolution followed by a 3×3 convolution.
- TDSConv2D: a 3×3 transposed convolution followed by a 3×3 depthwise separable convolution.

The quantitative comparison of the evaluated UpS over the respective encoder architecture and precision data type is reported in Table 3. From the obtained results, we determine TDSConv2D to be the best up-sampling block since it is able to guarantee almost-real-time frequency performances over both 32-bit floating points and 8-bit integer architectures while showing the lowest RMSE. Moreover, two important aspects can be inferred from Table 3:

- The use of depthwise separable convolutions guarantees a boost of the frame rate in a range from 150% up to 200% with respect to classical convolutions.
- The transposed convolution layers achieve a comparable speed with respect to up-sampling operations (with a difference less than 2 fps) with an average RMSE improvement of almost 3%.

### 5.3. Input–Output Resolution

In this subsection, we analyze the behavior of the network while providing different input–output resolution pairs. We report in Table 4 the quantitative results where it is evidenced that both input and output dimensions remarkably affect the inference frequency on embedded devices. First of all, the input resolution has a stronger effect with respect to the output. This aspect can be recognized by observing, for example, the inference frequency boost (212% on the CPU and 339% on the TPU) while reducing the input resolution from 192×256 pixels to 96×128 pixels, keeping fixed the output resolution to 48×64. Vice versa, the resolution reduction in relation to the output depth map leads to a less noticeable inference frequency boost of 165% on average on the CPU and 146% on the TPU. Moreover, the final RMSE is not remarkably affected by the input–output image resolutions, with a maximum difference equal to 1.7 decimeters on the CPU and 8.6 on the TPU. Finally, from the reported analysis, we can conclude that the 96×128 input resolution and the 48×64 output resolution are the best trade-offs between speed and estimation error, and thus, are chosen for the proposed architectures.

### 5.4. Feasibility Study in Underwater Settings

In this section, we perform a feasibility study to understand the behavior of such models in the estimation of underwater depth maps. Due to the lack of underwater-labeled depth data publicly available for research, we compared state-of-the-art MDE methods (working at 32-bit precision) pre-trained on the terrestrial NYU Depth V2 dataset while evaluating their generalization capabilities by testing on the underwater dataset [26]. Other depth estimation methods designed for underwater environments such as [16,17,18,19] are not taken into account since their main task is the color restoration process without providing quantitative measurements of depth estimation error. We applied [36] as a pre-processing operation to the SQUID images in order to fix the color imbalance and recover their contrasts. Moreover, due to the different input–output image resolutions among the compared methods, we resized their predicted depth maps at the same 48×64 output size. The obtained results are reported in Table 5.

**Figure 3:**
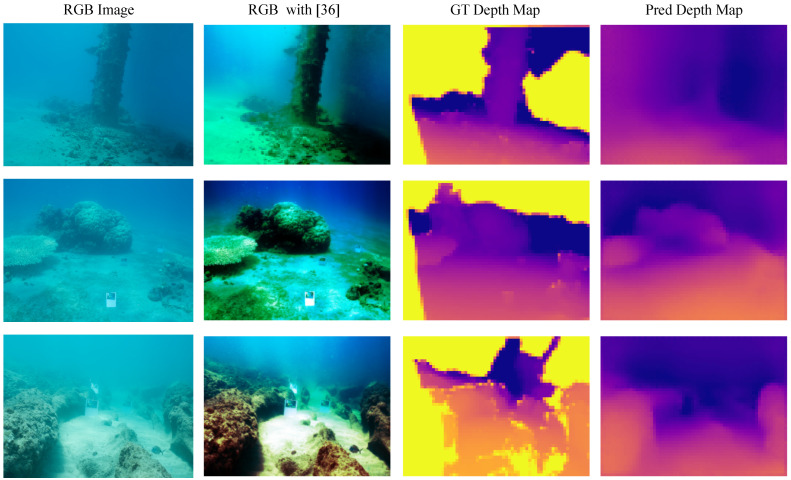
MobileNetV3${}_{S75}$ qualitative results over the SQUID dataset. The depth maps are all resized to the same image resolution and converted in RGB format with a perceptually uniform colormap (plasma-reversed) extracted from the ground truth, for a better view. Yellow ground truth depth-map pixels represent missing depth measurements.

We evaluated SQUID images only in those areas where depth distances are reported, without taking into account some missing depth distances present in ground truth images, a well-known behavior caused by the limitations of passive depth technologies in the acquisition of data. We can observe this fact in Figure 3 (third column), where missing depth measurements are represented as yellow pixels. As can be seen from the reported results, despite the high estimation error achieved by all the methods with respect to the terrestrial dataset, the proposed MobileNetV3${}_{S75}$ with only 1.1M of training parameters outperforms both [12] and [5] while obtaining the same RMSE and a higher $\delta_{1}$ to [11]. In addition, the proposed model achieves a boost on the inference frequency equal to ×4.7 with respect to [11] on the same benchmark hardware.

We assume that the underwater predictions are caused by a sensible statistical gap between the terrestrial training set and the underwater test set; we expect better results when a dedicated underwater dataset equivalent to the terrestrial one becomes available.

Moreover, to have a better understanding of the image preprocessing effects and a graphical comparison between the estimated and the ground truth underwater depth maps, we report in Figure 3 some dataset samples. Observing the images from left to right, we report the input samples (RGB Images) extracted from the SQUID dataset, the input samples after the prepossessing operation, and lastly, the ground truth (GT) and predicted depth (Pred) maps on which we apply a uniform colormap extracted from the ground truth samples. The reported depth maps show one of the advantages of MDE technology with respect to passive depth sensors, i.e., the estimation of filled depth maps (without missing measurements). Furthermore, by qualitatively comparing the predicted depths, we can see that the proposed solution is capable of handling the task by proposing blurred but reasonable depth estimations.

## 6. Energy Assessment

In this section, we provide an approach for measuring and analyzing the energy consumption of the proposed models during the inference phase. The objective of the study is to measure the trace of the current and the voltage to the device in which the inference is running and then find the total energy consumption (measured in Joule) of the test by numerically computing the integral, given *t* the duration of the experiment:

(4) $$E_{i}\left(t\right)=\int_{0}^{t}i\left(x\right)v\left(x\right)dx$$

**Figure 4:**
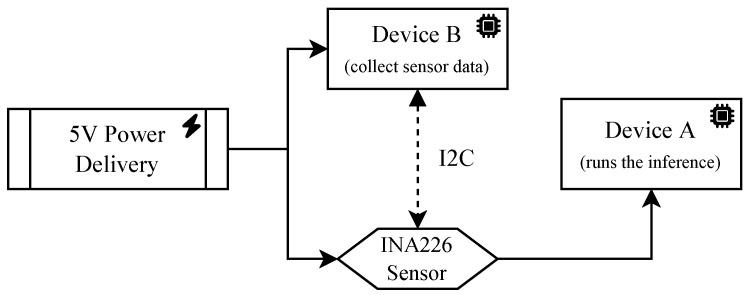
The conceptual diagram of the measurement setting. Device A performs the inference, while Device B collects the measurement data of the current and voltage from the INA226 sensor via the I2C protocol.

The measurements were taken by using, in conjunction, two devices and a voltage/current sensor (Figure 4). The first device (Device A) is the one in which the inference is run. We used a Raspberry Pi 3 and the Google Coral DevBoard. The second device (Device B), a Raspberry Pi 3, is connected via I2C protocol to a voltage/current sensor breakout board based on the Texas Instruments INA226. The current flowing to Device A passes through the sensor, which measures the voltage and current at the same time. The sensor has a sampling rate that is approximately near to 2000 Hz. Therefore, it allows for capturing the power behavior even for neural networks with FPS greater than 30 fps (standard real-time threshold value). In this particular case, we estimate to have ≈66 samples for every frame. We remark that the sampling rate is not constant, but it depends on the CPU activity.

The tests presented in this section are based on 100 sessions of 25 s in which we loaded each image from persistent storage and 100 sessions of 40 s where the entire dataset was loaded in RAM before starting the inference. Device B started recording the current and voltage measurements, and in parallel, the inference was started on Device A for the given amount of seconds.

### 6.1. Current and Voltage Footprint

**Figure 5:**
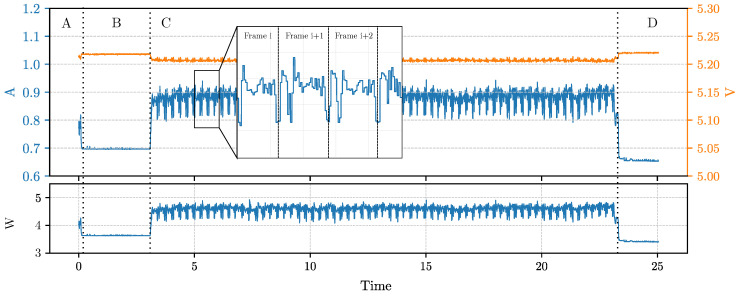
Trace of instant current and voltage obtained during the inference test for the model MobileNetV3${}_{LMin}$. In the figure, the current signal is zoomed in order to highlight the behavior of the current during the inference of the single frames. The figure shows phase A before running the benchmark script, phase B which is a sleep phase of 3 s, phase C in which the inference is performed continuously for 20 s and, finally, phase D where the system returns idle.

**Figure 6:**
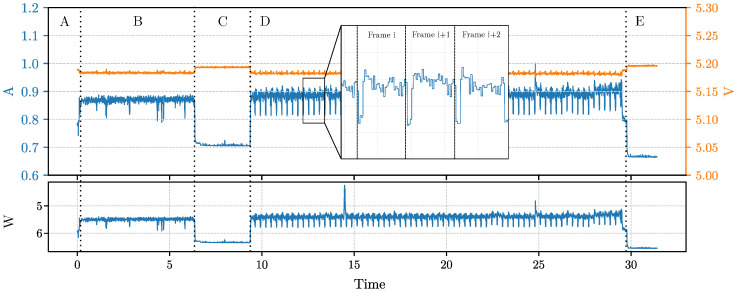
Trace of instant current, voltage and power obtained during the inference test for the model MobileNetV3${}_{LMin}$ but with the dataset entirely loaded in RAM (during phase B). In the figure, the current signal is zoomed in order to highlight the behavior of the current during the inference of the single frames. The figure shows phase A, before running the benchmark script, phase B, in which the dataset is entirely loaded in RAM, phase C, which is a sleep phase of 3 s, phase D, in which the inference is performed continuously for 20 s and, finally, phase E where the system returns idle.

The purpose of this section is to study the current and voltage traces of the inference process. We selected, as a test, the model which uses MobileNetV3${}_{LMin}$ as encoder and TDSConv2D as decoder (see the model with i=19 in Table 6). This was selected because the model in question has a low inference frame rate, thus allowing to clearly highlight its energy behavior during the inference of every frame. We ran two experiments in the first (Figure 5); we only performed the inference for 20 s, while in the second (Figure 6), we first loaded the entire dataset in RAM and then we performed the inference process. Figure 5 shows the traces of current (in blue) and voltages (in orange) during the entire test. We can easily distinguish from the graph the main phases of the procedure. Phase A comprehends the start of the test routine, while in phase B, we executed a process sleep of 3 s (we used the Python function time.sleep() in order to highlight in the charts when the inference process started); then, the C phase regards the inference, and finally, in the D phase, the device returns to the idle state. What we can observe is that, first of all, the sleep phase has an instant current absorption of 700 mA, which is slightly higher than the idle phase in which we have 680 mA. Then, during the inference, we can easily distinguish each analyzed frame. This is because the model runs at about 4.60 FPS. Every frame inference begins with a current drop of about 100 mA on average due to the passage to the next frame and the tensor allocation for the inference. The instant power consumption during the inference is, on average, 4.589 W.

Moreover, in Figure 6 we report the result of the same test while keeping the dataset entirely loaded in RAM during phase B. In this second test, the loading of the dataset in memory requires about 5.6 s (phase B); then, the script executes the sleep for 3 s (phase C) and the inference (phase D) for 20 s. Finally, the system goes idle in phase E. What we can observe from the traces is that the dataset load does not interfere with the actual power consumption of the inference. What can be noticed is that having the image already loaded in RAM, which can be the case of an image that is retrieved from a camera sensor, makes the power trace during inference more stable. This is because loading images from persistent storage is slow and requires a certain amount of waiting time, depending on the size of the image.

### 6.2. Models Inference Energy Consumption

In our setup, the model inference is a CPU-bound task, and it does not require interaction with storage or network, at least when the images to be processed are already loaded in RAM. Moreover, the classic inference with a model is not distributed, and it is a single-threaded operation. This means that during the inference, one CPU core will be used at one hundred percent of its time, independently from the model that it is used. However, different CPU operations have different energy consumptions. For example, the energy required for memory access depends on the type of data read or written [37]. Motivated by this idea, we studied which is the energy impact of the inference in every float32 model that we considered in this paper, assuming that images are directly loaded from RAM, which is the typical usage scenario when, for example, a camera is attached to the device.

For performing the energy assessment of the models, we conducted a series of 100 tests per model as the one presented in Figure 6. For each test *k* s.t. 1≤k≤100 and for each model *i* s.t. 1≤i≤34, we then computed the total energy consumption during the inference by using Equation (4), called $E_{i}^{k}\left(t\right)$. From there, the energy consumed per second is actually the average power requirement during the inference, for t=20, which is the duration of the experiment, can be defined as:

(5) $$P_{a_{i}}^{k}\left(t\right)=\frac{E_{i}^{k}\left(t\right)}{t}$$

We will omit the time *t* since it is fixed for every test. The average power requirement for the inference of model *i* is the average among all tests, and then:

(6) $$P_{a_{i}}=\frac{P_{a_{i}}^{k}}{k}$$

Table 6 shows the $P_{a_{i}}$ sorted for every model. We also reported the confidence interval based on the Student’s t-distribution with a *p*-value of 0.05. However, to give statistical significance to the mean value of the power, we conducted the ANOVA test with 34 degrees of freedom, that is, the number of models. The test, with a *p*-value =0.05, reports a significance of <0.001, F-Value =35.83 and $\eta^{2}=0.26$. This proves that some of the means are effectively different from others.

**Figure 7:**
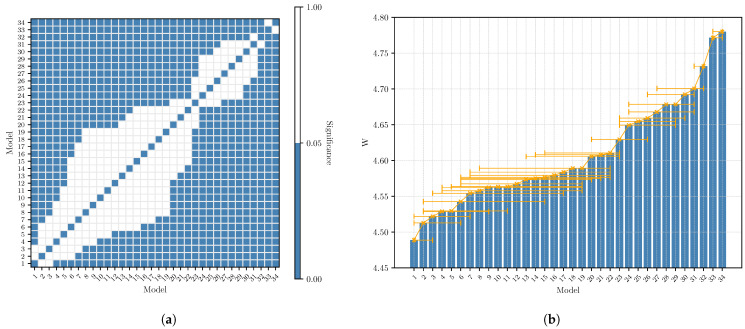
Results of the Student’s *t*-test on the average power consumption during inference ($P_{a_{i}}$) on every model *i*. The figures show that models with a similar architecture have comparable power consumption while others instead consume less or more power in a statistically significant way. The maximum difference in power consumption is in the order of 0.3 W. (**a**) Significance level of the Student’s *t*-test across all the models’ combinations. A blue square means that, given two models *i* and *j*, the difference between $P_{a_{i}}$ and $P_{a_{j}}$ is statistically significant (*p*-value =0.05). (**b**) The $P_{a_{i}}$ for every model listed in Table 6. The horizontal error bar has the same meaning as Figure 7a and describes the range in which the value is not statistically significant with respect to the others.

To clearly understand which means are statistically acceptable as different, we conducted a Student’s *t*-test between each possible model. The result of the test is shown in Figure 7, in particular, Figure 7a shows the significance of the *t*-test between each possible combination of two models, while Figure 7b report also the $P_{a_{i}}$ value for each model. What we can observe, also with the model data in Table 6, is that the power requirement for the inference is different only between specific models. In particular, we can notice how models which are based on the most energy-demanding architectures are both based on the “NNConv5” decoder, which is the only one based on 5 × 5 convolutional blocks, while the other has a 3 × 3 one.

We can conclude the energy assessment by giving some considerations about the results listed in Table 7. The first result is that the energy required for the inference ($P_{a_{i}}$) is not dependent on the inference time (that is $FPS^{-1}$) or on the floating-point operations (FLOPs), which the model requires. Actually, the Pearson correlation test reports a correlation of 0.319 (sign. <0.001, *p*-value =0.05) between the models’ FLOPs and the $P_{a_{i}}$ and −0.208 (sign. <0.001, *p*-value =0.05) between the FPS and the $P_{a_{i}}$. This suggests a weak correlation between the variables, in particular, a positive one for the FLOPs since a higher number of operations reduce the number of processed frame per second, therefore, it increases the time of CPU dedicated to the inference instead of preparing the model for the next frame. This also explains the negative correlation between $P_{a_{i}}$ and the FPS and the fact that the Pearson correlation between FPS and FLOPs is strong and negative (i.e., −0.812, sign. <0.001, *p*-value =0.05). The weak correlation between the power consumed ($P_{a_{i}}$) and the complexity of the model (FLOPs or FPS) is instead justified by the fact that what changes among the models is the type of operations that are carried out. Indeed, for example, models with i=34 and i=19 have a comparable complexity of about 470 M FLOPs; however, the models $P_{a_{i}}$ differ by 0.2 W. This is because the model with i=19, dealing with images of a bigger size, has to perform a higher number of memory access (and therefore a higher probability of cache miss) in which the CPU waits for the data to be retrieved and does not do any computation.

Finally, the results in Table 6 are sorted in terms of the mean $P_{a_{i}}$. In particular, we can observe that the first model (model with i=1) allows for saving about 6.48% of energy during the inference with respect to the last one (model with i=34). Instead, regarding the proposed models in Section 3, they are mapped to model with i=9 (Mob.NetV3${}_{S75}$), which represents the best trade-off between fps and RMSE, and the model with i=22 (Mob.NetV3${}_{LMin}$), which targets the 8-bit architecture. Since the energy assessment only targets the 32-bit architecture, we can observe that model i=9 also offers a relatively low energy consumption, which is 4.7% lower than the model with i=34.

## 7. Conclusions

In this work we proposed two variants of the MobileNetV3 encoder and a specifically designed decoder to tackle the MDE task in order to achieve real-time frequency performances on the embedded ARM CPUs and Edge TPUs. The method estimation capabilities are tested on the terrestrial NYU Depth V2 and underwater SQUID datasets. The obtained results demonstrate that the MobileNetV3${}_{S75}$ and the MobileNetV3${}_{LMin}$ models can be effectively considered solid candidates for the MDE task on the benchmark hardware, guaranteeing real-time frequency performances at the cost of a small increase in the estimation error on the terrestrial dataset. On the other side, the results in the underwater scenario show that in this peculiar environment, further studies and data are required in order to get reasonable monocular depth estimations. Despite the high error, our models are still producing better or on-par results with respect to the compared works with a sensible increase in inference speed.

Regarding the energy assessment, by performing 100 tests on the Google Coral Devboard IoT device, we proved (*p*-value = 0.05) that all the presented models have different power consumptions during the inference phase, with values ranging from 4.489 W (the least energy-demanding model) to 4.780 W (the most energy-demanding model), with a difference of 6.48% Moreover, by relying on the Pearson correlation, we showed that the energy required for inference does not strictly depend on the FPS or on the FLOPs (floating-point operations), which are characteristic of the specific model. This is because the energy consumption depends on the type of operations that are carried out during the inference.

Those findings will be a valuable baseline for future studies and advancements in the MDE field across embedded and mobile hardware. Further research will also be focused on embedded GPUs and the architectural and data changes required to get a robust and reliable depth estimation in underwater settings.

## Figures and Tables

**Table 1:** Depth estimation comparison of lightweight pre-trained encoders (32-bit float) with the TDSConv2D as an up-sampling block over the NYU Depth v2 dataset. The best results are in bold and the second best is underlined.

Method	RMSE↓	REL↓	$\delta_{1}↑$	CPU↑
	[dm]			[fps]
Eff.NetB0 [32]	6.01	0.179	0.728	4.4
NasNetMob. [33]	8.70	0.276	0.539	6.5
Mob.NetV1 [30]	**5.70**	**0.165**	**0.760**	8.8
Mob.NetV2 [31]	5.72	0.169	0.759	11.3
Mob.NetV3${}_{S}$ [28]	6.77	0.207	0.682	**26.2**
Mob.NetV3${}_{L}$ [28]	6.39	0.195	0.698	13.4

**Table 2:** Quantitative evaluation of the proposed models, the 32-bit floating point and the 8-bit integer precision, inferring on the ARM CPU and the Edge TPU.

Method	Type	RMSE↓	REL↓	$\delta_{1}↑$
		[dm]		
Mob.NetV3${}_{S75}$	32-bit	6.86	0.209	0.666
Mob.NetV3${}_{LMin}$	8-bit	11.54	0.429	0.412

**Table 3:** Comparison of different decoders with the proposed encoders tested over the NYU Depth v2 dataset.

Up-Sampling Block	MobileNetV3${}_{S75}$ (32-bit Float)				MobileNetV3${}_{LMin}$ (8-bit Int)			
	RMSE↓	REL↓	$\delta_{1}↑$	CPU↑	RMSE↓	REL↓	$\delta_{1}↑$	TPU↑
	[dm]			[fps]	[dm]			[fps]
UpConv2D	6.95	0.211	0.664	15.2	12.27	**0.360**	0.376	16.9
UpDSConv2D	7.02	0.212	0.660	**31.1**	13.55	0.399	0.283	**28.3**
NNConv5 [12]	7.69	0.236	0.608	6.8	21.86	0.654	0.030	9.5
TConv2D	6.88	**0.204**	**0.669**	18.1	17.63	0.836	0.218	17.5
**TDSConv2D**	**6.86**	0.209	0.666	29.6	**11.54**	0.429	**0.412**	26.8

**Table 4:** Comparison of the proposed encoder–decoder models with different input–output resolutions on the NYU Depth v2 dataset.

Resolutions		MobileNetV3${}_{S75}$ (32-bit Float)				MobileNetV3${}_{\mathrm{LMin}}$ (8-bit Int)			
Input	Output	RMSE↓	REL↓	$\delta_{1}↑$	CPU↑	RMSE↓	REL↓	$\delta_{1}↑$	TPU↑
		[dm]			[fps]	[dm]			[fps]
192×256	192×256	**6.38**	**0.194**	**0.687**	5.8	20.19	0.961	0.051	4.5
192×256	96×128	7.09	0.215	0.652	9.6	12.85	0.363	0.342	6.6
192×256	48×64	7.01	0.211	0.654	13.9	12.32	**0.314**	0.349	7.9
96×128	96×128	8.07	0.244	0.571	20.3	11.56	0.413	0.407	17.3
96×128	48×64	6.86	0.209	0.666	**29.6**	**11.54**	0.429	**0.412**	**26.8**

**Table 5:** Generalization capability comparison over the SQUID dataset [26].

Method	RMSE↓	REL↓	$\delta_{1}↑$	CPU↑	Parameters
	[m]			[fps]	[M]
DenseDetpth [5]	5.23	5.275	0.047	<1	42.6
FastDepth [12]	5.17	5.493	0.055	2.0	3.9
SPEED [11]	**4.49**	**4.732**	0.088	6.2	2.6
**Mob.NetV3${}_{S75}$**	**4.49**	4.956	**0.089**	**29.6**	1.1

**Table 6:** The list of all the tested models sorted by the $P_{a_{i}}$ parameter, which is the energy consumption of the Google Coral Devboard in Watts during the inference of the specified model.

							$P_{a_{i}}$ (*p* = 0.05) (W)		
*i*	Encoder	Decoder	In.	Out.	FPS	FLOPs	low.	mean	upp.
1	NasNetMob.	TDSConv2D	96 × 128	48 × 64	6.76	290.4M	4.468	4.489	4.510
2	Mob.NetV3${}_{S75}$	TDSConv2D	96 × 128	96 × 128	18.25	52.2M	4.501	4.513	4.525
3	Mob.NetV3${}_{S75}$	UpDSConv2D	96 × 128	48 × 64	36.44	47.1M	4.502	4.522	4.541
4	Eff.NetB0	TDSConv2D	96 × 128	48 × 64	4.49	267.8M	4.505	4.529	4.552
5	Mob.NetV3${}_{SHD}$	TDSConv2D	96 × 128	48 × 64	41.54	36.3M	4.509	4.529	4.550
6	Mob.NetV3${}_{LHD}$	TDSConv2D	96 × 128	48 × 64	16.88	119.9M	4.525	4.543	4.560
7	Mob.NetV3${}_{LMinHD}$	TDSConv2D	96 × 128	48 × 64	21.52	107.8M	4.533	4.554	4.575
8	Mob.NetV3${}_{S}$	TDSConv2D	96 × 128	48 × 64	28.07	46.7M	4.538	4.558	4.577
9	Mob.NetV3${}_{S75}$	TDSConv2D	96 × 128	48 × 64	31.14	39.2M	4.546	4.562	4.579
10	Mob.NetV3${}_{S75}$	TDSConv2D	192 × 256	192 × 256	4.89	206.0M	4.540	4.563	4.586
11	Mob.NetV3${}_{LMin}$	TDSConv2D	96 × 128	96 × 128	13.08	124.5M	4.541	4.563	4.586
12	Mob.NetV3${}_{LMin}$	TDSConv2D	192 × 256	192 × 256	3.41	497.9M	4.545	4.567	4.589
13	Mob.NetV3${}_{LMin}$	UpDSConv2D	96 × 128	48 × 64	18.88	127.0M	4.553	4.573	4.594
14	Mob.NetV3${}_{L50}$	TDSConv2D	96 × 128	48 × 64	25.17	55.2M	4.550	4.575	4.600
15	Mob.NetV3${}_{L}$	TDSConv2D	96 × 128	48 × 64	14.10	133.5M	4.558	4.576	4.595
16	Mob.NetV3${}_{S50}$	TDSConv2D	96 × 128	48 × 64	35.88	27.4M	4.561	4.579	4.598
17	Mob.NetV2	TDSConv2D	96 × 128	48 × 64	11.59	180.9M	4.566	4.584	4.601
18	Mob.NetV3${}_{L75}$	TDSConv2D	96 × 128	48 × 64	17.38	100.3M	4.565	4.589	4.613
19	Mob.NetV3${}_{LMin}$	TDSConv2D	192 × 256	96 × 128	4.60	476.0M	4.572	4.589	4.607
20	Mob.NetV3${}_{SMin}$	TDSConv2D	96 × 128	48 × 64	33.45	39.7M	4.580	4.606	4.632
21	Mob.NetV3${}_{LMin}$	TDSConv2D	192 × 256	48 × 64	5.50	454.6M	4.581	4.608	4.634
22	Mob.NetV3${}_{LMin}$	TDSConv2D	96 × 128	48 × 64	17.33	119.0M	4.582	4.611	4.639
23	Mob.NetV1	TDSConv2D	96 × 128	48 × 64	9.22	309.1M	4.602	4.629	4.656
24	Mob.NetV3${}_{SHD}$	TDSConv2D	96 × 128	48 × 64	27.38	67.8M	4.622	4.649	4.677
25	Mob.NetV3${}_{LMin}$	TConv2D	96 × 128	48 × 64	11.27	240.9M	4.629	4.655	4.680
26	Mob.NetV3${}_{LMin}$	UpConv2D	96 × 128	48 × 64	10.42	297.6M	4.638	4.659	4.681
27	Mob.NetV3${}_{S75}$	TConv2D	96 × 128	48 × 64	16.95	136.4M	4.642	4.668	4.694
28	Mob.NetV3${}_{S75}$	TDSConv2D	192 × 256	96 × 128	4.69	542.9M	4.650	4.678	4.706
29	Mob.NetV3${}_{S75}$	UpConv2D	96 × 128	48 × 64	15.87	185.5M	4.651	4.678	4.706
30	Mob.NetV3${}_{S75}$	TDSConv2D	192 × 256	48 × 64	6.79	410.7M	4.660	4.692	4.725
31	Mob.NetV3${}_{L200}$	TDSConv2D	96 × 128	48 × 64	5.47	440.0M	4.662	4.701	4.739
32	Mob.NetV3${}_{S200}$	TDSConv2D	96 × 128	48 × 64	5.74	485.1M	4.704	4.732	4.760
33	Mob.NetV3${}_{LMin}$	NNConv5	96 × 128	48 × 64	5.20	649.9M	4.746	4.771	4.797
34	Mob.NetV3${}_{S75}$	NNConv5	96 × 128	48 × 64	6.85	479.3M	4.750	4.780	4.810

**Table 7:** The Pearson correlation between models FLOPs, FPS and $P_{a_{i}}$. (**) The correlation is significant at the 0.01 level (2-tailed).

		FLOPs	*P* ${}_{a_{i}}$	FPS
**FLOPs**	Correlation	1	0.319 **	−0.812 **
	Sig. (2-tailed)		<0.001	<0.001
	N	3400	3400	3400
***P*** ** ${}_{a_{i}}$ **	Correlation	0.319 **	1	−0.208 **
	Sig. (2-tailed)	<0.001		<0.001
	N	3400	3400	3400
**FPS**	Correlation	−0.812 **	−0.208 **	1
	Sig. (2-tailed)	<0.001	<0.001	
	N	3400	3400	3400

## Data Availability

The code and the pre-trained weights are available at https://github.com/lorenzopapa5/code-2023-sensors (accessed on 30 January 2023).
